# Immunogenic Yeast-Based Fermentate for Cold/Flu-like Symptoms in Nonvaccinated Individuals

**DOI:** 10.1089/acm.2009.0310

**Published:** 2019-05-03

**Authors:** Mark A. Moyad, Larry E. Robinson, Edward T. Zawada, Julie Kittelsrud, Ding-Geng Chen, Stuart G. Reeves, Susan Weaver

**Affiliations:** ^1^ Department of Urology, University of Michigan Medical Center, Ann Arbor, MI.; ^2^ Embria Health Sciences, Ankeny, IA.; ^3^ Avera North Central Kidney Institute, Sioux Falls, SD.; ^4^ Department of Mathematics & Statistics, South Dakota State University, Brookings, SD.

## Abstract

***Background:*** The common cold has a profound impact on employee attendance and productivity. Seasonal influenza is responsible for approximately 200,000 hospitalizations and 36,000 deaths per year in the United States alone. Over-the-counter medication efficacy has been questioned, and seasonal vaccination compliance issues abound. Our previously reported randomized trial of an oral fermentation product found an adjuvant benefit for vaccinated individuals in terms of a significantly reduced incidence and duration of cold and flu-like symptoms.

***Methods:*** A concurrent 12-week, randomized, double-blind, placebo-controlled clinical trial of 116 subjects with no recent history of seasonal influenza vaccination was conducted. Participants received once-daily supplementation with 500 mg of a dried modified *Saccharomyces cerevisiae* oral fermentate (EpiCor) or placebo. Clinical outcome measurements included periodic interval-based in-clinic examinations and serologic analysis at baseline, 6 weeks, and 12 weeks. Participants utilized a standardized self-report symptom diary.

***Results:*** Subjects receiving the intervention experienced a statistically significant reduction in the incidence (*p* = 0.01), a nonsignificant reduction in duration (*p* = 0.10), and no impact on the severity (*p* = 0.90) of colds or flu-like symptoms, but a more favorable safety profile compared with subjects receiving placebo.

***Conclusions:*** This nutritional-based fermentate appeared to be safe and efficacious in a unique at-risk population and should receive more clinical research as a potential method to reduce the incidence of cold and flu-like symptoms, in individuals with and without a history of influenza vaccination.

## Introduction

The common cold and its impact on work place absenteeism is well recognized.^1^ It has become the third most common reason for physician office visits behind that of only hypertension and the recommended well-infant/child examinations.^2^ Seasonal influenza's impact on morbidity and mortality rates are more concerning. In the United States alone, an estimated 200,000 hospitalizations and 36,000 deaths are attributed each year to this virus.^3,4^ The Center for Disease Control (CDC) recently expanded the influenza immunization recommendations to include children beyond the age of 6 months and most adults.^3^

Compliance with vaccination recommendations has not mirrored the concerns of most established medical organizations, including the CDC.^3^ A recent report on health care workers in the United States demonstrated that only 33% had received the influenza vaccination,^5^ which generally reflects the rate of compliance reported by practitioners around the world.^6^ This may be a primary reason the public has yet to embrace the importance of the vaccine. Other direct and indirect reasons for the low compliance rate may include (1) a recently released report that most strains in the current 2007–2008 vaccine were ineffective in preventing the majority of the flu cases;^3^ (2) past perceptions that the vaccine was of minimal value;^3^ (3) recent reports of viral resistance to prescription medication;^7,8^ (4) ongoing evidence to suggest that many over-the-counter (OTC) preventive methods and medications to treat potential symptoms have no clinical value, or no exemplary clinical data, or safety issues;^9–11^ and (5) concerns over a mercury preservative in the existing vaccine supply.^3^ However, ample evidence exists to at least refute most of these controversial issues surrounding the efficacy and safety of vaccination.^12,13^

Regardless, other safe and clinically tested methods that can be utilized to improve the immune status of the general public would still seem to be of interest, which would include those individuals who choose not to comply with vaccine recommendations; those who delay their own access or do not have immediate access to the vaccine; or individuals in the well-documented 2-week maximal antibody-generating waiting period postvaccination.^3^

Additionally, the spectrum of cold and flu symptoms overlap,^14,15^ and an intervention that could impact the incidence of one or both of these conditions would again be another option in the ongoing search for effective OTC preventive items.

An oral immunogenic fermentation product (EpiCor^®^, Embria Health Sciences, Ankeny, IA) partially derived from *Saccharomyces cerevisiae* (*S*. *cerevisiae*) has already demonstrated the potential for adjuvant immune enhancement in a randomized, double-blind, placebo-controlled trial of vaccinated subjects for influenza.^16^ Significant reductions occurred in both the incidence and duration of cold and flu symptoms. This trial addressed and answered one of two primary questions with this once-daily OTC supplement intervention: the potential capacity to safely enhance an already effective conventional medicine or at least add something novel during the most susceptible time of the year to cold and flu-like conditions. In this current clinical trial, we report the findings of the second concurrent trial and primary question that needed to be answered and construed: the ability to display some immunogenic potential when utilized as a sole agent in individuals who chose not to be vaccinated for seasonal influenza.

## Materials and Methods

All methods listed in this section have been previously well described and were identical to our previous adjuvant trial,^16^ with the exception of the nonvaccinated status requirement for this current study. The age range was 18–76 years, and subjects were living in a metropolitan area of the rural Midwest. Individuals had to be in good general health, as reflected by a Charlson comorbidity score of 0 or 1,^17^ and via a standard basic history and physical examination by the clinical and research staff. Exclusion criteria are noted in Table 1.

**Table 1.** Exclusion Criteria Utilized Before Randomization in the Cold and Flu Study of the Oral Fermentate-Based Product Compared to Placebo

History of an influenza vaccination in the past 12 months
Diagnosed, managed, or treated immune abnormality
Current use of any immunosuppressive prescription or over-the-counter medication such as azathioprine, cyclosporine, and steroids
Current use of any antiviral medication including amantadine, oseltamivir, rimantadine, and zanamivir
HIV positive
ALT, AST, BUN, and/or creatinine laboratory values greater than 2 times the upper limit of normal
Females who are pregnant, breastfeeding, or who are planning to become pregnant during the study period
History of substance abuse
Moderate to severe co-morbidity or concomitant disease or condition (Charlson score of 2 or greater)
Allergies to yeast or any yeast-derived products
Environmental allergies requiring medication or allergy-based injection therapy
Vitamin, mineral, or nutrient deficiency that requires supplementation
Herbal or supplemental preparation use in any form or formulation such as echinacea, vitamin C, or zinc
Unable or unwilling to comply with the study protocol, including ingesting the study supplement or placebo, regular blood sampling, and completing the study diary
Current participation in another clinical research investigation of any kind

HIV, human immunodeficiency virus; ALT, alanine aminotransferase; AST, aspartate aminotransferase; BUN, blood urea nitrogen.

This 12-week, randomized, double-blind, placebo-controlled trial was conducted during the acute period of the year for cold and flu seasonal symptoms (January through March). Healthy individuals without a recent history of vaccination for seasonal flu (influenza) giving informed consent (*n* = 116) were screened to determine baseline standardized laboratory values including complete blood count, complete metabolic profile, and other serologic parameters. Subjects were randomized to one of two groups: 500 mg of the daily, oral fermentate product (EpiCor, *n* = 58) or placebo (n = 58) for 12 consecutive weeks. The placebo capsule was of an identical appearance, odor, and weight compared to the active intervention. Participants were instructed to ingest medications with the first meal of the day.

Subjects attended the research institute clinic at weeks 0 (baseline), 6, and 12, and were required to record cold and flu-like symptoms at home in a modified standardized diary provided by the research center.^18^ An overview of the diversity of the symptoms provided in this diary was provided in a previous publication.^16^ Symptoms in the diary were rated from 0 (no symptoms) to 10 (most severe symptoms) and included the following: headache, general aches/pains, fatigue, weakness, nasal stuffiness, nasal drainage, sore throat, cough, hoarseness, chest discomfort, chills, fever, and miscellaneous or other, which had to be specified by the participant.

Each periodic clinical visit included: a standard history and physical examination, serologic sampling, vital signs (blood pressure, heart rate, temperature, and weight), on-site completed Short Form 36,^19,20^ and reviewed and summarized diary information. The clinical study was approved by the Institutional Review Board for Avera Health (Sioux Falls, SD).

Common cold was clinically defined as an upper respiratory tract infection of viral etiology consisting of one or more of the following symptoms: cough, generalized malaise, headache, hoarseness, low-grade fever, nasal drainage, nasal stuffiness, and sore throat.^3,14,15^ Influenza-like symptoms were clinically defined as a respiratory tract infection of viral etiology and acute onset, more severe than the common cold, and consisting of one or more of the following symptoms: chest discomfort, fever of 102°F–105°F, myalgia, nonproductive cough, prominent headache, rhinitis, and sore throat.^3,14,15^ Cold and flu-like symptoms could clinically overlap or occur simultaneously. The incidence of cold or flu-like symptoms was defined as the number of clinical occurrences reported during the entire 12-week study period. Duration of symptoms was defined as the number of consecutive illness days, and severity was also recorded and defined by a scale from 0 to 10 (least to most) as described by the self-report diary. The primary objective of the clinical trial was to determine whether a once-daily dose of the nutritional intervention would reduce the incidence, duration, and/or the severity of the common cold or influenza-like symptoms in healthy human subjects with no recent history of seasonal influenza vaccination. The statistical analysis software used by the statistician was the R program (2.9.0), which can be reviewed and obtained from www.r-project.org Final statistical analysis on the outcomes utilized two-way analysis of variance with EpiCor and placebo as treatment factor, and all symptoms as another factor. Analysis of covariance was also utilized when adjusting for covariates.

## Results

Baseline characteristics for the EpiCor and placebo group are shown in Table 2.

**Table 2.** Baseline Characteristics of the Intervention and Placebo Group

*Baseline characteristic*	*Intervention (*n* = 58)*	*Placebo (*n* = 58)*
Age (mean ± SD)	37.1 (±13.5)	39.6 (±13.0)
Age range (years)	18–94	20–71
BMI (mean ± SD)	26.9 (±5.8)	27.0 (±4.2)
Gender (% female)	57%	60%
Race (% white)	97%	97%
Smoking status—never/past (%)	83%	85%
Smoking status—current(%)	17%	15%

BMI, body–mass index.

No statistical significance between baseline characteristics of either group was identified.

The intervention significantly (*p* = 0.01) reduced the incidence of the common cold or flu-like symptoms compared to placebo. A mean of 1.32 (95% confidence interval [CI] 1.25–1.39) versus 1.51 (95% CI 1.37–1.65) clinical events occurred between the intervention and placebo groups, and this result remained significant regardless of the separate or combined baseline status parameters. The intervention had a greater impact on reducing the overall risk or incidence of 10 of the 11 specific symptoms compared to placebo, with the exception of weakness. Duration was nonsignificantly (*p* = 0.10) reduced from 4.25 (3.54–4.96) to 3.59 symptom days (95% CI 3.14–4.03) compared to placebo, and this result was again similar, regardless of baseline status. Duration symptoms were also reduced compared to placebo for 9 of the 11 parameters, with the exception of chills and chest discomfort. Severity score was not impacted by the intervention compared to the placebo (*p* = 0.90), which was 3.57 (95% CI 3.26–3.89) to 3.60 (95% CI 3.3–3.89). Fever was not impacted compared to placebo for incidence, duration, or severity, and the event rate was low overall (16 events compared to 14 events).

No abnormalities were found with any of the laboratory serologic parameters when comparing the intervention at baseline, to the intervention at 12 weeks, or when comparing intervention to placebo. The intervention significantly reduced systolic (*p* = 0.04) and diastolic (*p* = 0.01) blood pressure compared to placebo by 4 and 3 mm Hg, respectively.

The compliance rate (number of capsules consumed over the study period) in the intervention versus the placebo group was similar, with approximately 90% of capsules consumed. The rate of reporting any adverse event(s) was 29% for EpiCor and 48% for the placebo group, which is a significant difference (*p* = 0.02). There were a total of 3 dropouts during the trial, 1 in the placebo and 2 in the supplement group. Dropout was due to the lack of subject compliance with the protocol. None of the dropouts was for medication-related issues (intervention or placebo).

## Discussion

Seemingly never-ending myriad untested OTC options for the prevention and relief of cold and flu-like symptoms exist,^10^ and in the United States approximately $3 billion annually is spent on cold preparations alone.^21,22^ Another estimated $1 billion goes toward filling unnecessary antibiotics prescriptions for these viral etiologies.

The ongoing concern by the U.S. Food and Drug Administration over nonefficacious or unsafe remedies should continue to result in the need to enforce more stringent research criteria for commercial availability and health claims.^9–11^ This has been highlighted recently in studies that are challenging some long-standing and widely available untested and unproven OTCs available to consumers in the United States with simple and cost-effective home remedies.^23,24^ These results should continue to generate thoughts about evidence-based medicine or simply the lack of evidence in some areas of the OTC market.

Additionally, despite nationally based educational efforts, a large segment of the population continues to forgo the seasonal influenza or other effective vaccinations.^6,12,25^ Knowledge of this documented discrepancy, its consequences, an appreciation of the spectrum of flu-like signs and symptoms and a lack of OTC data were sufficient reasons for our research team to design and implement this unique trial.

*S*. *cerevisiae* and/or products resulting from its fermentation with various substrates seem to be an appropriate choice for immune maintenance because of a long and notable clinical history of safety and clinical efficacy.^26,27^ For example, one of the largest randomized trials of dietary selenium supplementation for cancer prevention demonstrated overall significant reductions in total cancer incidence, colorectal, prostate, and lung carcinoma. This trial utilized a modified 500 mg *S*. *cerevisiae* tablet that included 200 μg of selenium, as opposed to selenium by itself.^28^ A more recent large-scale randomized trial of a combination low-dose nutritional supplement intervention included 100 μg of selenium that was also *S*. *cerevisiae*-derived, and researchers found a significant reduction in the risk of cancer in men, including prostate cancer.^29,30^ No changes in hormone levels, prostate-specific antigen, or insulin-like growth factor 1 were noted despite these clinical benefits.^30^ These past observations, along with the observations from our two trials, suggests that perhaps other mechanisms exist whereby risk reduction is achieved.

EpiCor was developed by Embria Health Sciences, LLC, of Ankeny, IA, and is classified as a dietary supplement.^31^ It consists of *S cerevisiae*, grown under anaerobic and nutritional stress, in association with the nutrients and metabolites present in the fermentation broth, and in combination desiccated into a powdered form.^16^ Over the last 60 years, a commercial feed additive product for farm animals only, based on this proprietary technology, has been utilized to enhance immune function and to prevent disease. The human-modified version of this product (EpiCor) has recently been subjected to multiple laboratory safety, stability, and efficacy investigations. It has demonstrated general and specific anti-inflammatory properties and potential immune support in humans with the stimulation of B-lymphocytes and natural killer cells, ^32^ and significantly increased salivary immunoglobulin A levels from a preliminary open label study of 22 adults before this trial was initiated (data on file). The same yeast species utilized in our trial may harbor a unique immune-modulating capacity because it is also utilized as the principal harvesting system for the current hepatitis B vaccine (HBV).^33^ HBV is prepared via harvesting surface antigen of hepatitis B from cell cultures of recombinant strains of *S*. *cerevisiae*. Taken together, the objective and subjective data, and the direct and indirect evidence from *S*. *cerevisiae*-based technology were of an appropriate credibility to attempt some initial stage of immune therapy in a real-world setting.

EpiCor contains a series of macronutrients including fatty acids, such as oleic acid, which is found in olive oil, for example, and also a variety of soluble and insoluble dietary fibers. It contains almost the entire series of B-vitamins and minerals, but it is also unique in terms of its concentration of phytosterols and phenolic compounds such as resveratrol. Many of these individual compounds are at least the recipient of beneficial laboratory and clinical studies in medicine and immunology,^34–38^ but in the oral fermentate they may synergistically garner an immune-modulating potential that may have some clinical application.

The *S*. *cerevisiae*-derived fermentate in this trial was not only safe but seems to provide some positive or no cardiovascular changes including blood pressure reductions. Similar to the ongoing paradigm with prescription preventive medicines, a dietary supplement, in our opinion, needs to have demonstrated some measure of safety, especially no cardiovascular issues, before being considered in this OTC category.

The results of this trial preliminarily espouse the previous observations and data on this modified *S*. *cerevisiae* fermentation product.^16^ Incidence was moderately reduced between 10% and 20%; however, a total of 11 of the 12 symptoms decreased with this intervention. Duration of symptoms also decreased, which translated into an almost entire day of symptomatic reduction when cold and flu-like symptoms occurred. Thus, in total, the immune-protective properties seem consistent and noteworthy.

The overall strengths of this current study, especially for a dietary supplement, are also numerous and noteworthy. Based on strict and accepted methodological scoring systems utilized to analyze past clinical trials,^39^ our trial fulfilled the majority of these criteria, which included (1) the large number of participants; (2) randomization of group assignment; (3) maintenance of the double-blind or treatment allocation concealment; (4) baseline similarities of the groups; (5) a withdrawal/dropout rate or narrow confidence intervals unlikely to cause bias; (6) blinding of the outcome assessors; (7) and a predefined primary outcome measurement and result completely reported. In our opinion, these are features, which are not commonly observed in OTC product studies. In addition, the strict exclusion and inclusion criteria, and the real-world setting of utilizing a product in a nonvaccinated milieu further establish the integrity of the observations. The financial cost to conduct such a clinically robust trial is a further testimony to the investigative team and the manufacturer of this product. Our research team also found that once-a-day dosing is a benefit in terms of simplicity and compliance issues.

Limitations of this clinical trial should also receive attention. More frequent clinical visits, albeit costly, would have allowed for closer follow-up and more precise serologic observations. The standardized diary is an imperfect system of measure, but was reviewed with each visit. Additional immunologic plasma, serum, urine, and imaging studies could have further enhanced the accuracy of the trial, including the duration and severity data. However, it should be reiterated that the monitoring of primarily symptoms in the case of colds and flu-like conditions remains the accepted standard for primary outcome measures utilized in conventional medical prescription drug and vaccine trials.^40^ It would have also been advantageous to have information on workplace or household contacts who have been vaccinated that could potentially provide a herd immunity effect or a potential immunologic shield for a clinical trial participant. Randomization should have provided balance in terms of this concern, and it was reassuring to find no significant difference in baseline health characteristics among the intervention and placebo groups. Nevertheless, our recruitment methods failed to attract a diversity of participants in terms of minority group participation. This needs to be addressed and amended in future trials. Finally, an intent-to-treat design was not utilized, similar to the previous trial,^16^ but only 3 participants dropped out of this study, and the overall methodology along with this compliance rate and consistency in the findings from the past and current trial should be sufficient, in our opinion, to ensure confidence in the results with this intervention compared to placebo.

## Conclusions

In conclusion, this randomized trial demonstrated that a modified *S*. *cerevisiae*-based oral immunogenic fermentate taken once daily is safe and significantly reduced the incidence, and nonsignificantly reduced the duration of cold and flu-like symptoms. This is now the second randomized, double-blind, and placebo-controlled trial to date to demonstrate the potential for this product to improve clinical endpoints in an otherwise healthy population, regardless of vaccine history. These studies should potentially also serve as at least minimal criteria, in our opinion, for the type of research needed to establish credibility in the OTC market for cold and flu-like symptom prevention.
